# Macular Pigment Reflectometry: Developing Clinical Protocols, Comparison with Heterochromatic Flicker Photometry and Individual Carotenoid Levels

**DOI:** 10.3390/nu13082553

**Published:** 2021-07-26

**Authors:** Pinakin Gunvant Davey, Richard B. Rosen, Dennis L. Gierhart

**Affiliations:** 1 College of Optometry, Western University of Health Sciences, Pomona, CA 91766, USA; 2 Department of Ophthalmology, New York Eye and Ear Infirmary of Mount Sinai, Icahn School of Medicine at Mount Sinai, New York, NY 10029, USA; rrosen@nyee.edu; 3 ZeaVision LLC, Chesterfield, MO 63005, USA; dgierhart@zeavision.com

**Keywords:** macular pigment optical density, MPOD, lutein, zeaxanthin, carotenoids, heterochromatic flicker photometry, age related macular degeneration, biomarker

## Abstract

The study was designed to: (1) Analyze and create protocols of obtaining measurements using the Macular Pigment Reflectometry (MPR). (2) To assess the agreement of MPOD measurements obtained using the heterochromatic flicker photometry (MPS II) and MPR. (3) To obtain the lutein and zeaxanthin optical density obtained using the MPR in the central one-degree of the macula. The measurements were performed using the MPR and heterochromatic flicker photometry. The MPR measurements were performed twice without pupillary dilation and twice following pupillary dilation. The MPR measurements were performed for a 40-s period and the spectrometer signal was parsed at different time points: 10–20, 10–30, 10–40, 20–30, 20–40, and 30–40 s. The MPR analyzes the high-resolution spectrometer signal and calculates MPOD, lutein optical density and zeaxanthin optical density automatically. The MPR-MPOD data was compared with MPPS II-MPOD results. The MPR-MPOD values are highly correlated and in good agreement with the MPS II-MPOD. Of the various parsing of the data, the data 10–30 interval was the best at obtaining the MPOD, lutein, and zeaxanthin values (8–12% coefficient of repeatability). The lutein to zeaxanthin ratio in the central one-degree of the macula was 1:2.40. Dilation was not needed to obtain the MPOD values but provided better repeatability of lutein and zeaxanthin optical density. MPR generates MPOD measurements that is in good agreement with MPS II. The device can produce lutein and zeaxanthin optical density which is not available from other clinical devices.

## 1. Introduction

The National Institute of Health has taken a bold and much needed step known as the Precision Medicine Initiative [1,2,3]. Precision Medicine, also known as Individualized Medicine, is defined as “an emerging approach for disease treatment and prevention, taking into account individual variability in genes, environment, and lifestyle for each person” [1]. Although this initiative is primarily focused on cancer and its management, the lessons learnt, and creative methodologies employed are indeed applicable to other disease states [1,2]. Moroi et al. [4] have described three guiding principles of precision medicine in glaucoma that can have application to other conditions. The principles summarized are (a) phenotyping disease states with new definitions and latest technology; (b) discovery, validation and understanding of biomarkers; and (c) revamping epidemiological studies to discover and validate the risk factors that affect the onset and progression of the disease.

To this accord the macular pigment optical density (MPOD) is a clinical biomarker that is altered in various disease states like age related macular degeneration [5,6,7,8,9,10,11,12,13,14,15]. The MPOD score is not only associated with many ophthalmic conditions but also with systemic diseases like diabetes [16,17]. The level of MPOD is also closely related to the carotenoid levels seen in the brain [18,19] making it a helpful biomarker which is associated with various cognitive functions [20,21,22,23,24,25,26,27]. Importantly, the levels of MPOD can be successfully enhanced in healthy eyes and in disease states using oral supplementation with carotenoids, making it a modifiable risk factor [5,6,7,8,9,10,11,12,13,14,15,16,28,29,30,31,32,33,34,35,36,37].

The MPOD can be measured in-vivo using both subjective [5,6,8,10,11,25,26,28,38,39,40,41,42,43,44,45,46] and objective techniques [47,48,49,50,51,52,53,54,55,56,57,58,59,60]. The subjective technology heterochromatic flicker photometry is far more readily available in measuring MPOD [5,6,8,10,11,25,26,28,38,39,40,41,42,43,44,45,46]. Although it is proven to be reliable and repeatable it is prone to the difficulties that affect any psychophysical tests [45]. The heterochromatic flicker photometry is an indirect measure of the MPOD and requires both patient and practitioner training. The heterochromatic flicker photometry is a time-consuming procedure (approx. 5 min per eye) and is not capable of measuring the MPOD levels in the disease states where individuals have poor visual acuity and ocular fixation [5,45]. Possibly due to these reasons, large randomized controlled trials like the (Age-Related Eye Disease Studies (AREDS) trials did not measure MPOD [61]. The AREDS-2 trial [61] did show that the carotenoid supplementation with lutein and zeaxanthin indeed favored treatment, particularly in those that had low serum levels at baseline. Thus, MPOD could indeed be an important biomarker in managing patients with ocular diseases particularly macular degeneration.

There is indeed interest in obtaining MPOD rapidly using more objective techniques. The MPOD can be measured objectively using imaging technology like the dual wavelength autofluorescence [47,53,54,55,57,58,59] and resonance Raman imaging techniques [56]. These devices provide MPOD objectively in living humans and are gaining momentum in the research community. The dual wavelength autofluorescence technique [47,53,54,55,57] is described in literature and available in few research centers but is not FDA approved in the USA. Raman imaging, although described in literature, is also not commercially available [56]. These devices are either standalone in measuring MPOD or are coupled with imaging technologies like optical coherence tomography which are expensive. Thus, the heterochromatic flicker photometer remains the most commonly used clinical device to measure MPOD due to its small footprint and it being relatively inexpensive [5,6,8,10,11,25,26,28,38,39,40,41,42,43,44,45,46].

The Macular Pigment Reflectometry (MPR) [48,49,50,51,52] is an objective technique that can measure MPOD in a very short duration. The MPR analyzes the light reflected from the retina using a high-resolution spectrometer to calculate the MPOD. Additionally, the reflection spectra of the carotenoids present in retina are different and with certain assumptions and predetermined calculations the lutein and zeaxanthin optical density are also measured by MPR. Prior publications [48,49,50,51,52] using research prototypes have shown the MPR to be a promising new technology in measuring MPOD related parameters. The MPR is also shown to be capable of tracking changes in MPOD with oral supplementation of carotenoids. 

The current clinically available technologies that measure MPOD both subjective and objective are not capable in providing an estimate of lutein and zeaxanthin optical densities. By obtaining new biomarkers like lutein and zeaxanthin optical densities allows for a more tailored carotenoid supplementation therapy than the “one size fits all” strategy that is currently utilized. Thus, if the MPR technology is commercialized and is indeed successful in obtaining repeatable MPOD and carotenoid optical density it would indicate a substantial progress in the field of precision medicine. 

In the present study we evaluate a near commercial prototype of MPR (ZeaVision LLC, St. Louis, MO, USA). The overall goal of the study as a first attempt was to assess the MPR technology clinically. The aims of the study were: (1) To assess and analyze the data obtained by MPR and to create a clinical protocol that can be utilized in future studies and in clinic. This was accomplished by assessing the MPR signal at various time intervals and assess what duration of data provided the best repeatability of MPR measurements of MPOD, lutein and zeaxanthin optical densities. (2) To compare the MPOD measurements obtained using the heterochromatic flicker photometry and MPR. This is important as the heterochromatic flicker photometry is the current gold standard of MPOD measurements and it is important to assess new technology with existing technology. (3) To evaluate and compare the measurements of lutein optical density and zeaxanthin optical density obtained using the MPR in the central one-degree of macula. 

## 2. Material and Methods

Participants were evaluated at the Western University of Health Sciences, College of Optometry. The study was approved by the institutional review board at Western University of Health Sciences, Pomona, California, USA, and conducted in accordance with the tenets of the Declaration of Helsinki (IRB 17/IRB/025). Participants signed a consent form, were adults of at least 18 years of age, and were deemed to have good ocular health during a comprehensive ocular evaluation by an optometric physician in the last year. Participants additionally underwent a study-specific ophthalmic evaluation. This included visual acuity evaluation, ocular health evaluation of both anterior and posterior segment using slitlamp biomicroscope, fundus evaluation, and photography. The evaluation of retina was performed after pupillary dilation which was achieved using tropicamide 0.5% ophthalmic solution. Participants with any retinal pathology, glaucoma, or history of surgery that could influence measurements were excluded. All participants had a logMAR visual acuity of +0.0 (20/20) or better.

A total of 30 eyes of 30 participants was included in the study. The mean age of study participants was 30.7 years (standard deviation 6.9; range 23–48 years), the age was normally distributed (Kolmogorov–Smirnov D = 0.14; *p* > 0.10). Of the participants included in the study, eight were males and 22 were females. The mean age of males was 27.9 years and mean age of females was 31.8 years which was not statistically different (F-test f = 1.77; *p* = 0.3) 

### 2.1. Measurements Using Heterochromatic Flicker Photometry

The MPOD measurements were obtained using the heterochromatic flicker photometry MPS II (Electron eye Technology, Cambridge UK) [38,39,40,41,45,46] which is a subjective psychophysical device that estimates MPOD. The central macula and fovea contain xanthophylls and have a characteristic absorption spectrum. The target viewing distance was set to infinity using an optical system and central stimulus had a one-degree angular subtense. The central stimulus is generated from blue, green, and white LEDs on a background luminance of 250 cd/m^2^. The MPS II displays two light stimuli of different wavelengths (green 530nm and blue 465 nm). The light stimuli alternates between a blue light of short wavelength which is perceived as a flicker. The blue light is absorbed by the macular pigments, and a green light is not absorbed by the macular pigment.

The patient was instructed to fixate at the central target. The subject was instructed to press the button when observing a flicker in the target. The first step was to determine the threshold and was accomplished by the presentation of first five targets. The testing continued, and the machine altered the radiance of blue versus the radiance of green until a constant target was perceived as having no flicker. This was marked the as the lowest point on the graph. The test continued with increase in blue radiance until a flicker was perceived and tested three times consecutively at various increments of blue radiance. The MPOD measured was adjusted to account for the normal age-related yellowing of the lens and a final MPOD was reported in density units (du).

**Figure 1:**
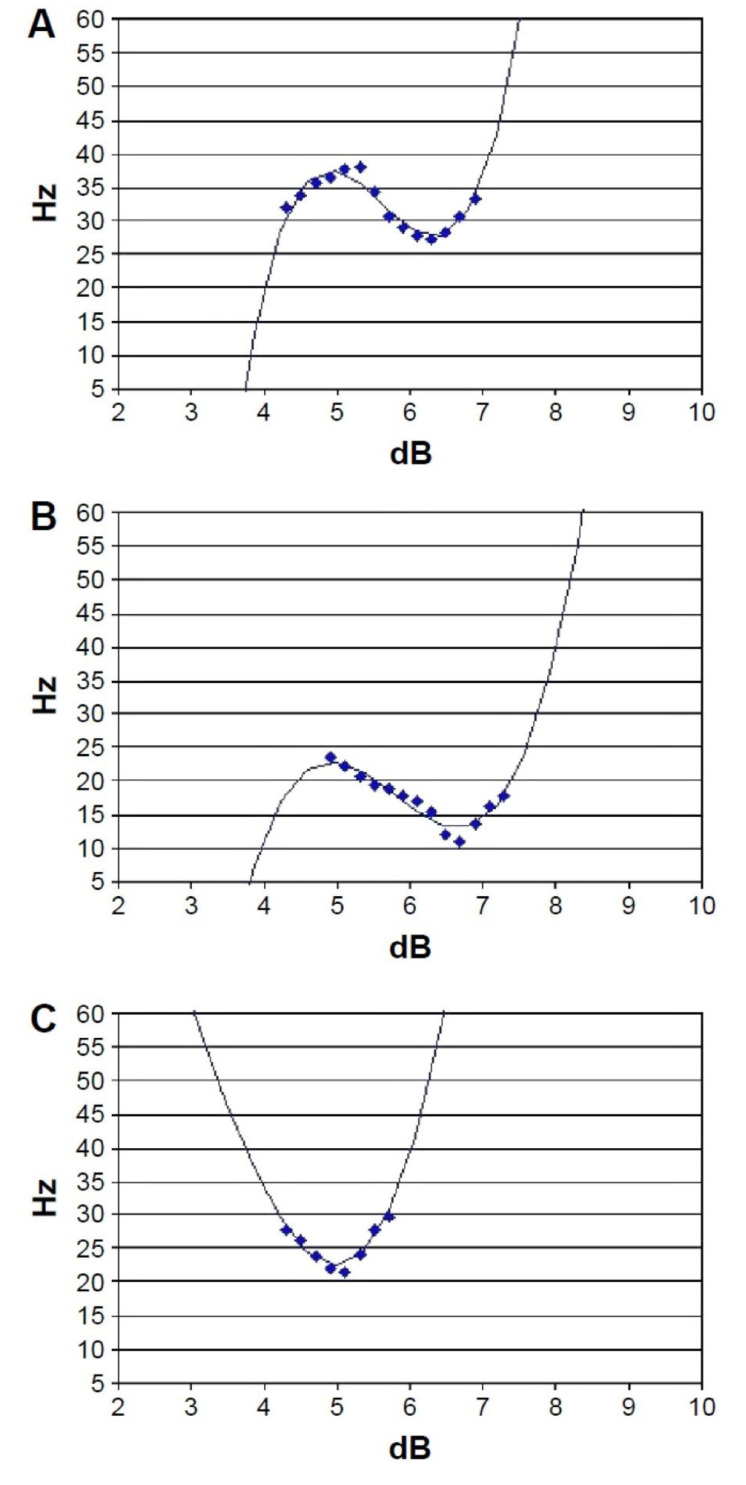
The output of subjects performing the MPSII. The panels (**A**–**C**) show various types of MPOD (macular pigment optical density) output graphs that are deemed acceptable, and have a lowest point and were considered acceptable.

The eye undergoing testing was randomized for each participant and only one eye data of an individual was used for the study purposes. The testing time for each eye was approximately 2 min. The device provided an automatic quality index of output if the results were acceptable, borderline acceptable, or not acceptable. The results included a graph and a value of the MPOD. The graphs were additionally evaluated for reliability using a set criterion [45]. A good test shows the following characteristics: (1) smooth downward slope, (2) single lowest point, and (3) three upward raise. Figure 1 provides examples of MPS II output. The MPS II measurements were performed twice, and measurements were averaged to obtain the mean MPOD values. All participants had an acceptable quality index of results and met the visual inspection of reliability.

### 2.2. Measurements Using Macular Pigment Reflectometry

The measurements were performed using the MPR which is a prototype (ZeaVision LLC, St. Louis, MO, USA). The measurement of MPOD was based on a quantitative model analysis of light reflected at various layers in the eye with different absorbers in between. Prior publications [48,49,50,51,52,53,54] have explained the optical design and mathematical equations and have provided preliminary data using the MPR. The MPR employs a technique that can successfully measure lutein and zeaxanthin optical density in vivo in humans [48,49,50,51,52,53,54]. The MPR uses a quartz halogen source along with a series of filters (to prevent UV damage) along with Badal system to project a controlled full spectrum (400 to 800nm) light spot onto the retina of the patient. The illumination pupil acts as a semi-circular mask which determines the shape of the illumination beam as it enters the pupil of the patient’s eye. The retinal stop is used for forming an illumination beam that defines a circular illumination field of one-degree at the retina (i.e., approximately a 300 µm diameter). This illumination spot is also the visual reference which serves as the fixation target for the patient. The light reflected from the retina is collected and analyzed by a high-resolution spectrometer and a mathematical function is fit to estimate various parameters of interest. The automated integrated software of the MPR analyses the signal from the retina using a spectrometer and produces measures of MPOD, lutein, and zeaxanthin optical density. Lens optical density is directly measured and is included in the algorithm [48,49,50,51,52,53,54]. The lens model used in the current implementation of the MPR is the van de Kraats model which returns two components for lens absorption Kyoung and Kold. The young component is a “base” measurement while the old represents the ongoing age sensitive component of the lens. This parameter was not analyzed in the current study as the study sample consisted of young individuals who had no media opacities or age-related lens changes. The spectral absorption of lutein and zeaxanthin has a similar shape, but the zeaxanthin curve is approximately 10 nm shifted towards the longer wavelengths. The resolution of the spectrometer used in this device is 5.8 nm and sufficiently sensitive to measure lutein and zeaxanthin optical densities as shown in prior papers [48,49,50,51,52,53,54]. It is important to note that the zeaxanthin optical density includes both the levels of zeaxanthin and meso-zeaxanthin which is an isomer of zeaxanthin. The spectral absorption characteristics of zeaxanthin and meso-zeaxanthin are very similar and the MPR is unable to distinguish between the two carotenoids.

The measurements by MPR in theory can be performed indefinitely and it is up to the user to specify the measurement duration. The prior laboratory research used one-second measurement time that was performed after alignment. The disadvantage for such a short measurement time is greater chance of an error if patient fixation is questionable. In the present study the measurements were performed for 40 s. The first 10 s of the data signal was discarded as there is a bleaching of the photoreceptor pigment during that time and using that signal would lead to erroneous MPOD calculations. Measurements were performed a total of four times; twice under undilated condition and twice with pupillary dilation. A two-minute break was provided after each measurement and the subjects were requested to take the chin off the chin rest to simulate a more real-life measurement variability. The MPR data that was collected for 40 s was parsed into six-time stamped intervals i.e., 10–20, 10–30, 10–40, 20–30, 20–40 and 30–40 s. The MPR software analyzes the various time-stamped intervals automatically to generate MPOD, lutein and zeaxanthin optical density. The measurements obtained both without and with pupillary dilation were respectively analyzed as described above.

### 2.3. Statistical Analysis

The distribution of normality was assessed using the Kolmogorov-Smirnov test. The coefficient of repeatability was assessed for the MPSII MPOD measurements and the MPR MPOD measurements. The difference between the values between the first and second measurements were analyzed for statistical significance using a paired samples *t*-test for both undilated and dilated conditions. Pearson correlation coefficient were calculated between the first and second MPOD, lutein and zeaxanthin optical density obtained using the MPR device. Pearson correlation coefficient was utilized to evaluate correlation between the MPOD values obtained using the MPS II and the MPR devices, respectively. Agreement was assessed using the Bland-Altman plots. Linear regression analysis was utilized to derive an equation to predict MPS II MPOD from the MPR-MPOD data. 

## 3. Results

### 3.1. Measurement with MPS II Heterochromatic Flicker Photometry

The mean MPOD values of first measurement was 0.455 (SD 0.16) and the repeat measurement was 0.463 (SD 0.16). The coefficient of repeatability of MPOD measured using the MPS II heterochromatic flicker photometry was 12.8%. The mean MPOD of first and second measurements was 0.459 (SD 0.15) and utilized for comparisons and predictive regression analysis model.

### 3.2. Measurement Obtained Using Macular Pigment Reflectometry 

The Table 1 and Table 2 provides the MPOD measurements obtained using the Macular Pigment Reflectometry (MPR) under undilated and dilated conditions respectively for both the first and second measurements. The various time stamped intervals evaluated are 10–20, 10–30, 10–40, 20–30, 20–40 and 30–40 s. The difference between the first MPOD measurement and the second MPOD measurement under undilated and dilated condition was not significantly different the data obtained from all six-time stamped intervals (paired samples *t*-test *p* > 0.05 respectively).

**Figure 2:**
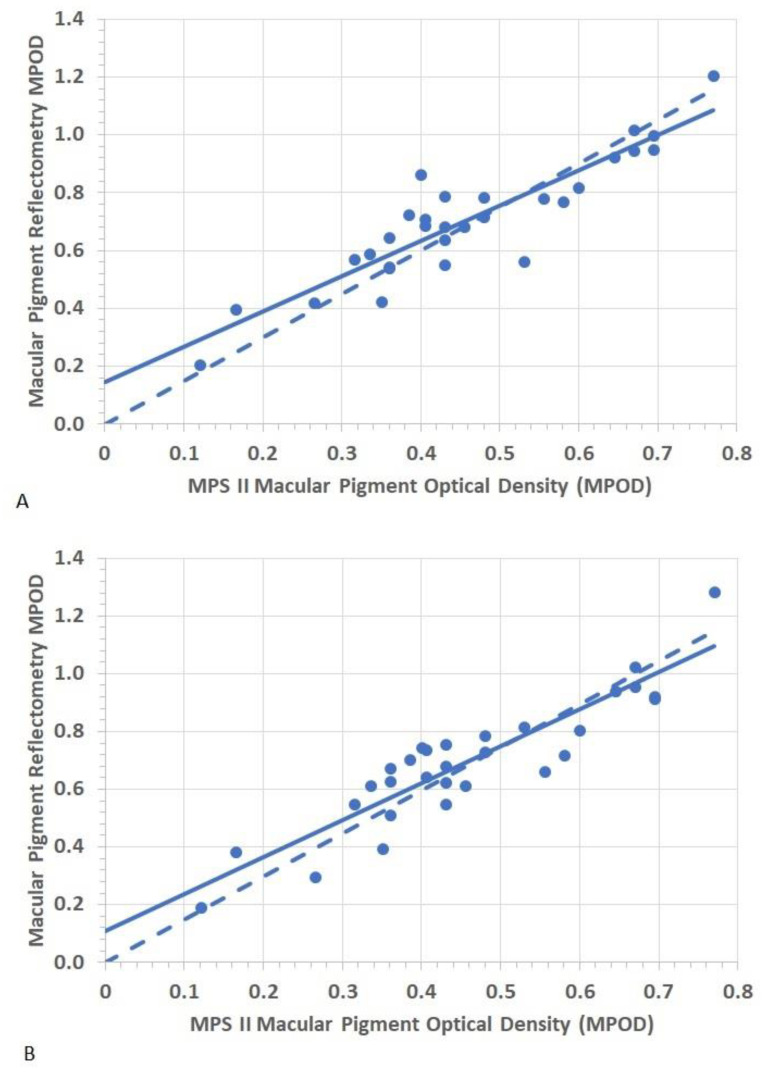
Shows bivariate linear regression analysis between the MPR- MPOD for 10–30 s time stamped interval data and MPS II-MPOD. The panel-(**A**), represents MPR-MPOD obtained undilated (r = 0.906) and panel-(**B**) represents MPR-MPOD obtained with dilated pupils (r = 0.910). The solid line is the regression line, and the dashed line is the zero-intercept, slope of one line.

The coefficient of repeatability on average was greater when MPOD measurements were performed using the MPR under undilated conditions compared to the dilated conditions. The Table 3 provides the mean MPOD obtained using the MPR device with and without pupillary dilation. Comparing the MPR-MPOD values, we find that the mean MPOD obtained in normal undilated pupils was not significantly different than the mean MPOD measured with pupillary dilation for all six-time stamped intervals (paired samples *t*-test > 0.05 respectively). There was a strong correlation between the mean MPSII-MPOD values and the mean MPR -MPOD values obtained in both undilated and dilated pupillary conditions which ranged from r-statistic 0.91–0.93 (See Table 4). To analyze the association further visually, we plotted the bivariate regression analysis between MPR-MPOD obtained both undilated and under dilated pupillary condition at 10–30 s time stamped interval with MPS II MPOD (see Figure 2). The regression slope is very close and similar to the zero intercept, slope of one indicating good association and agreement between the two MPOD measures. 

### 3.3. Agreement between MPS II-MPOD and MPR-MPOD Measurements 

Table 5 provides the Bland-Altmann agreement analysis for the MPS II-MPOD and the MPR-MPOD when assessed in both with and without pupillary dilation. The difference between mean values of MPSII- MPOD and MPR-MPOD which is called the “Bias” and the 95% limits of agreements are also reported for measurements obtained at all six-time stamped intervals. Although the correlation between the MPS II-MPOD and the MPR-MPOD is excellent (see Table 4), the agreements as assessed by Bland-Altmann analysis shows there is a systematic bias between the measurements obtained by the two devices. The mean difference ranges from 0.241 to 0.248 when measured in undilated normal pupillary condition and 0.239 to 0.244 when MPR-MPOD is measured with pupillary dilation. Given that the mean difference between MPS II-MPOD and MPR-MPOD is very consistent across all six-time stamped intervals, adjusting the raw MPR-MPOD score by 0.243 should yield better agreement between devices. 

### 3.4. Predicting MPS II-MPOD Score from MPR-MPOD Measurements

The MPS II device is commonly available in clinics that measure macular pigment optical density. Physicians are used to seeing an MPS II MPOD values. To aid in physicians being able to transition into new technology it will be welcome if MPR could generate a predicted MPS II-MPOD score. Given the mean difference between MPS II-MPOD and MPR-MPOD is very consistent, values are highly correlated (see Table 4) and the limits of agreement are narrow (see Table 5) one could accurately predict one from another. To this accord we utilized the linear regression equation using the MPS II-MPOD and the MPR-MPOD. The shortest time-stamped interval of MPR data that generated the highest coefficient of repeatability was used in the generation of the correction factor. The time stamped interval of 10–30 s under dilated pupillary condition generated the best coefficient of repeatability (see Table 1 and Table 2). The equation 1 given below can be utilized to predict the MPS II MPOD from the MPR-MPOD data.

*Predicted* MPS II-MPOD = +0.7011 (MPR-MPOD) − 0.0339 *R*^2^ = 0.85 (1)

*Predicted* MPS II-MPOD is the data or results likely to be generated by the heterochromatic flicker photometry and MPR-MPOD is macular pigment optical density obtained by macular pigment reflectometry. 

### 3.5. Measurements of Lutein and Zeaxanthin Optical Density Using the Macular Pigment Reflectometer 

The Table 6 and Table 7 provides the mean lutein optical density measures obtained twice without and with pupillary dilation. The Table 8 and Table 9 provides the zeaxanthin optical density measures obtained twice without and with pupillary dilation. As seen from the Table 6 and Table 8 there is a small but statistically significant difference between the first and second measure of lutein optical density and zeaxanthin optical density when obtained through physiological undilated pupillary conditions in some time stamped intervals. The lutein optical density and zeaxanthin optical density measurements obtained with pharmacologically dilated pupil was not significantly different between first and second measurement (Table 7 and Table 9).

As seen from lutein and zeaxanthin optical density measurements in Table 6, Table 7, Table 8 and Table 9 the amount of lutein in the central 1-degree of the macula is lower than the amount of zeaxanthin. The Table 10 provides the mean values of lutein and zeaxanthin optical density in males and females. The mean lutein optical density was 0.232 (SD = 0.14 range 0.04 to 0.57). The mean zeaxanthin optical density was 0.520 (SD = 0.22; range 0.10 to 1.10). The mean levels of lutein and zeaxanthin optical density was not significantly different between males and females (one way ANOVA *p* = 0.65 and 0.45 respectively). The median ratio of lutein to zeaxanthin is 1:2.40. Correlating the lutein optical density to zeaxanthin optical density we find that the higher levels of zeaxanthin were associated with lower levels of lutein, however this association was very weak and did not reach statistical significance (Pearson correlation r = −0.32; *p* = 0.08). 

## 4. Discussion

Prior studies [49,50,51,52,53] have evaluated the laboratory slit lamp-based MPR unit and have reported values of MPOD, lutein and zeaxanthin. They have also reported that MPR was sensitive to measuring increased MPOD when carotenoid supplements were consumed for a given period [50]. This study utilized a near commercial prototype of MPR in measuring the MPOD and the lutein and zeaxanthin optical density. The goal of this study was to evaluate the measurements obtained using the MPR and compare it with the MPS II heterochromatic flicker photometry values and to create clinical protocols that would be useful for future experiments. To fulfill these goals, we decided to obtain measurements twice using the MPR normal physiological pupils and twice after pharmacological dilation of pupils. The MPR measurements were performed for a total of 40 s. The first 10-s of signal was not utilized as the signal might be changing due to the bleaching of pigment in photoreceptors. The time stamped interval of 10–30 s is ideal at providing the MPOD, lutein and zeaxanthin optical density values. This conclusion was arrived at after looking at various measures of coefficient of repeatability, difference between first and second measurements, correlation between first and second values and agreement with MPS II -MPOD. It appears that additional 10 s of data that is signal from 10–40 s times stamped interval did not significantly improve the values over values obtained from 10–30 s. 

When measuring MPR-MPOD, pupillary dilation did not appear necessary; the mean values obtained under both undilated and dilated pupillary conditions were not significantly different. The correlation coefficient of MPS II-MPOD and MPR-MPOD was very high under both dilated and undilated pupillary conditions. Therefore, we recommend that pupillary dilation is not necessary if physicians are only interested in the MPR-MPOD values. Evaluating the lutein and zeaxanthin optical density measurements we found that the values obtained without pupillary dilation were slightly more variable as assessed by repeat measurements (see Table 6, Table 7, Table 8 and Table 9). Pupillary dilation is not essential for lutein and zeaxanthin optical density measurements obtained at 10–30 s time stamped data as the first and second measurements are not significantly different. However, there was the higher correlation and better coefficient of repeatability, between first and second measurements of lutein and zeaxanthin optical density obtained with pupillary dilation. The reason for more reliable data obtained with pupillary dilation is possibly due to the better signal quality with dilated pupils. The small pupils size occurs due to the bright halogen light of MPR and it is likely to cutoff some of the light from entering the eye and some reflected light from retina. Thus, we recommend that both lutein and zeaxanthin optical density be obtained when pupils are pharmacologically dilated. 

Comparing the MPOD measurements obtained using the two devices MPS II and MPR we found that the limits of agreement were narrow but there were a consistent mean difference of 0.243 between the measures. The MPR-MPOD values were consistently greater than the MPS II-MPOD data. Given that both these devices are measuring the same parameter, there needs to be a recalibration or adjustment of the MPR-MPOD values. A “bias correction” and recalibration should be performed which will provide greater agreement between technologies. 

The coefficient of repeatability is a very important metric in assessing clinical suitability of a device. The value also can be utilized to assess what level of change can be measured accurately by a device and not a random function of “noise” or chance. The MPOD measured using the MPS II device has a coefficient of repeatability of 12.8% which is consistent with prior reports [45]. The coefficient of repeatability for the MPOD measurements obtained using the MPR device was 8%. This would suggest that repeatability of MPOD measurements obtained by MPR is about 60% better than that obtained by MPS II which may produce clinically significant improvement in the sensitivity and accuracy of MPOD measurement. However current study used relatively small sample size and the true benefits of MPR should be evaluated in larger sample size trial. 

A prior publication by van der Veen et al. [51] compared MPOD values obtained using the heterochromatic flicker photometry (HFP) and a prototype MPR device in 19 ocular healthy individuals. They found that the correlation was good between the two with correlation coefficient r = 0.72 [51]. Our study found the correlation coefficient was excellent and in the range of 0.94–0.98. There are differences between present study and prior work [51]. The present study the data acquisition time was approximately 30 s, compared to 1-s acquisition time in prior work [51]. The difference in duration of measurement, greater data sampling and averaging of 30 -second data may explain the difference in coefficient of correlation observed between HFP and MPR in the two studies. Most interestingly, the MPR technology [48,49,50,51,52,53,54] has the ability to measure lutein and zeaxanthin optical density in vivo, which is currently not available using any other technology. This may offer significant clinical advantages as applied to precision and individualized medicine. It could help answer fundamental questions and enhance our understanding of both physiological and pathological states. It is important to remember that the zeaxanthin optical density includes both the levels of zeaxanthin and meso-zeaxanthin as their spectral reflections are currently indistinguishable from each other. The lutein optical levels measured in the present study were lower than zeaxanthin levels centrally. The lutein to zeaxanthin ratio 1:2.24 measured closely matches results that have been previously published from histological experiments and laboratory slit lamp based MPR [49,50,51,52,53,62]. The coefficient or repeatability of lutein and zeaxanthin optical density was 12% and 9% respectively. 

There have been other types of reflectometry techniques in literature. Delori et al., [60] compared their reflectometry techniques with both heterochromatic flicker photometry and autofluorescence technique to measure MPOD. Delori et al. [60] found mean MPOD as 0.23 du whereas we had a mean MPOD of 0.70 du. The reproducibility estimates were also different 22% in Delori et al.’s work whereas we found 8% coefficient of repeatability [60]. The reflectometry technique used in Delori et al., study [60] is based on different principles of measurements than the reflectometry used in current study. They use a two-step technique to measure MPOD centrally, then peripherally and the subtraction of two provides MPOD. Whereas the MPR used in the present study only requires one measurement and mathematical modelling and quantitative analysis of light reflected from various layers of the eye as they pass different absorbers. This along with difference in sample size, ethnogeographical differences in study population may be responsible for different mean MPOD estimates seen in the two studies.

The MPR needs 10 s of photopigment bleaching time whereas the quantitative auto-fluorescence technique photopigment beaching is performed for 20 s. This may in part be due to the larger area of MPOD measurement and it uses a different type of wavelength and luminance of light source. If the MPOD measurements of quantitative autofluorescence technique and MPR -MPOD is compared it is probably best done for 20–40 s time stamped interval and may possibly yield better correlation between these two techniques. 

HPLC is the laboratory gold standard in measuring carotenoids and the heterochromatic flicker photometry is the current widely used clinical standard of MPOD measurements [63]. HPLC leads to tissue destruction, and the absorption spectrum of lutein and zeaxanthin depends upon kind of solvent used during the procedure. Olive oil is the best solvent to be used with HPLC that provides the absorption spectra of lutein and zeaxanthin in retina which is 452 and 463 nm respectively [63]. The MPR can successfully measure lutein and zeaxanthin as its spectrometer resolution (5.8 nm) is approximately twice more than the difference in peak absorption spectra of lutein and zeaxanthin in retina (11 nm). 

AMD remains a leading cause of blindness worldwide and low MPOD levels have been associated with AMD [5,6,7,8,9,10,11,12,13,14,15]. The damage to the photoreceptors and the retinal pigment epithelium in AMD is irreversible. Numerous advances have been made in the understanding of the disease and therapy for exudative or neo-vascular AMD using anti-vascular endothelial growth factor therapies have been enormously successful. However, the more widespread non-exudative or dry AMD form still has no proven therapy with the exception of vitamin supplementation [61]. The AREDS-2 trial [61] evaluated the effects of carotenoid supplementation on progression of AMD. The AREDS trial did not measure the MPOD but performed serum analysis of carotenoids. The sub-analysis favored the use of carotenoids lutein and zeaxanthin in treating individuals with AMD particularly individuals that had lower levels of serum carotenoids at baseline benefited the greatest. It is difficult to know the exact reasons why MPOD was not measured by the AREDS trial [61], but one could speculate, that the subjective nature of heterochromatic flicker photometry, time required for measurements, degree of variability in measurements and the inability to perform measurements reliably in patients with sub-optimal vision like intermediate stage AMD may be some of the reasons. The MPR device used in the present study can rapidly, directly, and objectively measure MPOD, with better repeatability than MPS II heterochromatic flicker photometry would be possible solution to the problems of clinical measurements of MPOD.

It is now generally accepted that the MPOD levels can be augmented through increased dietary intake of carotenoids or by taking them through oral supplements [5,6,7,8,9,10,11,12,13,14,15,16,28,29,30,31,32,33,34,35,36,37]. The MPOD measurement serves as a measurable physiological biomarker which can be monitored accurately over time. There are no perfect MPOD measuring devices as each have their advantages and disadvantages. HPLC whereas is the laboratory “gold standard” and can measure individual carotenoids separately leads to tissue destruction and cannot be used in vivo. The MPR not only provides MPOD but also levels of lutein and zeaxanthin optical density that gives additional information which was not available to physicians. This represents a substantial upgrade to the MPOD related measurement techniques currently available. Examining the levels of lutein and zeaxanthin optical density we find there is a wide range with high inter-individual variability. The MPR’s ability to measure individual component of MPOD that is lutein and zeaxanthin optical density would allow for tailoring the carotenoid supplementation to what is needed than a “one size fits all” current regimen. This brings us a few steps closer to precision medicine in treatment and management of retinal disease. Future studies are needed to evaluate long term reproducibility as well are inter-practitioner variability of parameters output by MPR. 

## 5. Conclusions

This study shows that using the clinical protocols described, the MPR device provides a repeatable MPOD, lutein and zeaxanthin optical density values which closely matches heterochromatic flicker photometry the current clinical gold standard of MPOD measurement. The in-vivo measurement of lutein and zeaxanthin optical density in the central one-degree of macula using the MPR devices matches closely with published literature. The MPOD, lutein, and zeaxanthin optical density provided by the MPR would be a useful measurement in the pursuit of precision medicine for various ophthalmic and systemic diseases. 

## Figures and Tables

**Table 1:** Mean MPOD obtained using the Macular Pigment Reflectometry without pupillary dilation.

Time-Interval	Mean Undilated MPOD Measure 1	Mean Undilated MPOD Measure 2	Paired Samples *t*-Test *p*-Value	Coefficient of Repeatability
10–20 s	0.699	0.702	0.87	18%
10–30 s	0.705	0.702	0.82	13%
10–40 s	0.704	0.703	0.92	11%
20–30 s	0.709	0.704	0.66	11%
20–40 s	0.708	0.707	0.86	9%
30–40 s	0.702	0.712	0.37	11%

**Table 2:** Mean MPOD obtained using the Macular Pigment Reflectometry with pupillary dilation.

Time-Interval	Mean Dilated MPOD Measure 1	Mean Dilated MPOD Measure 2	Paired Samples *t*-Test *p*-Value	Coefficient of Repeatability
10–20 s	0.691	0.697	0.55	11%
10–30 s	0.695	0.701	0.50	8%
10–40 s	0.696	0.699	0.70	8%
20–30 s	0.700	0.706	0.44	9%
20–40 s	0.700	0.701	0.90	9%
30–40 s	0.700	0.699	0.94	11%

**Table 3:** Mean Macular Pigment Reflectometry MPOD (macular pigment optical density) obtained with and without pupillary dilation.

Time Interval	Undilated MPOD Average of First and Second Measurement	Dilated MPOD Average of First and Second Measurement	Paired Samples *t*-Test *p*-Value
10–20 s	0.702	0.694	0.90
10–30 s	0.702	0.698	0.75
10–40 s	0.703	0.697	0.68
20–30 s	0.704	0.703	0.62
20–40 s	0.707	0.701	0.57
30–40 s	0.712	0.699	0.75

**Table 4:** Pearson correlation coefficient between mean MPS II-MPOD (macular pigment optical density) and mean MPR-MPOD obtained with and without pupillary dilation.

Time Interval	R-Statistic Mean Undilated MPR-MPOD and Mean MPS II MPOD	R-Statistic Mean Dilated MPR-MPOD and Mean MPS II MPOD
10–20 s	0.91	0.92
10–30 s	0.92	0.92
10–40 s	0.92	0.93
20–30 s	0.92	0.93
20–40 s	0.92	0.93
30–40 s	0.92	0.93

**Table 5:** Agreement between MPS II-MPOD (macular pigment optical density) and MPR-MPOD measurements with and without pupillary dilation.

Time Interval	Bland-Altmann Agreement Analysis between MPS II-MPOD and MPR-MPOD			
	MPR-MPOD-Undilated Pupils		MPR-MPOD-Pupils Dilated	
	Bias	95% Limits of Agreement	Bias	95% Limits of Agreement
10–20 s	0.241	0.066 to 0.415	0.234	0.039 to 0.429
10–30 s	0.244	0.073 to 0.415	0.239	0.049 to 0.429
10–40 s	0.245	0.047 to 0.413	0.238	0.055 to 0.421
20–30 s	0.248	0.070 to 0.425	0.244	0.058 to 0.429
20–40 s	0.248	0.076 to 0.420	0.242	0.062 to 0.422
30–40 s	0.248	0.075 to 0.423	0.240	0.065 to 0.416

**Table 6:** Lutein optical density measurements obtained through physiological undilated pupillary conditions.

Time Interval	Undilated Attempt-1	Undilated Attempt-2	Paired Sample *t*-Test *p*-Value	Coefficient of Repeatability	Pearson Correlation r-Statistic
10 to 20 s	0.261	0.296	0.18	27%	0.72
10 to 30 s	0.255	0.3023	0.08	29%	0.71
10 to 40 s	0.248	0.307	**0.01**	25%	0.78
20 to 40 s	0.248	0.310	**0.01**	24%	0.77
30 to 40 s	0.254	0.298	0.05	23%	0.79
20 to 30 s	0.241	0.311	**0.01**	27%	0.74

Bold values represent a paired samples *t*-test *p* value being significant *p* < 0.05.

**Table 7:** Lutein optical density measurements obtained through pharmacologically dilated pupillary conditions.

MPR-Time Interval	Dilated Attempt-1	Dilated Attempt-2	Paired Sample *t*-Test *p*-Value	Coefficient of Repeatability	Pearson Correlation r-Statistic
10 to 20 s	0.271	0.209	0.67	20%	0.74
10 to 30 s	0.223	0.232	0.44	12%	0.92
10 to 40 s	0.224	0.243	0.38	23%	0.73
20 to 40 s	0.231	0.236	0.72	13%	0.91
30 to 40 s	0.230	0.238	0.66	19%	0.82
20 to 30 s	0.226	0.224	0.91	17%	0.83

**Table 8:** Zeaxanthin optical density measurements obtained through physiological undilated pupillary conditions.

Time Interval	Undilated Attempt-1	Undilated Attempt-2	Paired Sample *t*-Test *p*-Value	Coefficient of Repeatability	Pearson Correlation r-Statistic
10 to 20 s	0.521	0.525	0.82	21%	0.87
10 to 30 s	0.528	0.496	0.06	18%	0.91
10 to 40 s	0.516	0.505	0.64	23%	0.86
20 to 40 s	0.532	0.508	**0.04**	12%	0.96
30 to 40 s	0.529	0.499	0.10	19%	0.90
20 to 30 s	0.535	0.505	**0.03**	14%	0.95

Bold values represent a paired samples *t*-test *p* value being significant *p* < 0.05.

**Table 9:** Zeaxanthin optical density measurements obtained through pharmacologically dilated pupillary conditions.

Time Interval	Dilated Attempt-1	Dilated Attempt-2	Paired Sample *t*-Test *p*-Value	Coefficient of Repeatability	Pearson Correlation r-Statistic
10 to 20 s	0.521	0.542	0.11	14%	0.94
10 to 30 s	0.516	0.524	0.33	.09%	0.98
10 to 40 s	0.518	0.523	0.60	10%	0.97
20 to 40 s	0.521	0.519	0.81	10%	0.97
30 to 40 s	0.521	0.509	0.28	12%	0.96
20 to 30 s	0.519	0.530	0.28	10%	0.97

**Table 10:** Comparison of age, lutein and zeaxanthin optical density values of participants.

	Age	Lutein Optical Density	Zeaxanthin Optical Density
Males	27.9	0.251	0.469
Females	31.8	0.225	0.538
Average	30.7	0.232	0.520
